# Individual protomers of a G protein-coupled receptor dimer integrate distinct functional modules

**DOI:** 10.1038/celldisc.2015.11

**Published:** 2015-06-16

**Authors:** Nathan D Camp, Kyung-Soon Lee, Jennifer L Wacker-Mhyre, Timothy S Kountz, Ji-Min Park, Dorathy-Ann Harris, Marianne Estrada, Aaron Stewart, Alejandro Wolf-Yadlin, Chris Hague

**Affiliations:** 1 Department of Genome Sciences, University of Washington School of Medicine, Seattle, WA, USA; 2 Department of Pharmacology, University of Washington School of Medicine, Seattle, WA, USA

**Keywords:** GPCR, proteomics, scribble, syntrophin, pharmacology, PDZ domain

## Abstract

Recent advances in proteomic technology reveal G-protein-coupled receptors (GPCRs) are organized as large, macromolecular protein complexes in cell membranes, adding a new layer of intricacy to GPCR signaling. We previously reported the α_1D_-adrenergic receptor (ADRA1D)—a key regulator of cardiovascular, urinary and CNS function—binds the syntrophin family of PDZ domain proteins (SNTA, SNTB1, and SNTB2) through a C-terminal PDZ ligand interaction, ensuring receptor plasma membrane localization and G-protein coupling. To assess the uniqueness of this novel GPCR complex, 23 human GPCRs containing Type I PDZ ligands were subjected to TAP/MS proteomic analysis. Syntrophins did not interact with any other GPCRs. Unexpectedly, a second PDZ domain protein, scribble (SCRIB), was detected in ADRA1D complexes. Biochemical, proteomic, and dynamic mass redistribution analyses indicate syntrophins and SCRIB compete for the PDZ ligand, simultaneously exist within an ADRA1D multimer, and impart divergent pharmacological properties to the complex. Our results reveal an unprecedented modular dimeric architecture for the ADRA1D in the cell membrane, providing unexpected opportunities for fine-tuning receptor function through novel protein interactions *in vivo*, and for intervening in signal transduction with small molecules that can stabilize or disrupt unique GPCR:PDZ protein interfaces.

## Introduction

G-protein-coupled receptors (GPCRs) are a primary target for the development of novel therapeutics to treat disease. Traditionally, molecules directed towards GPCRs compete with the endogenous ligand for the orthosteric binding site. Unfortunately, biochemical similarities of closely related GPCR ligand binding pockets can hinder drug selectivity and narrow therapeutic index. Proteomic technology has provided opportunities to circumvent this technological barrier in GPCR drug discovery [1, 2]. In addition to heterotrimeric G-proteins, GPCRs can selectively interact with various accessory proteins, such as β-arrestins, multi-functional proteins that ubiquitously temper GPCR function [3], or one of the~260 PDZ (postsynaptic density protein, Drosophila disk large tumor supressor, zonula occludens-1 protein) domain containing proteins encoded in the human genome, which typically associate with PDZ ligands located in the GPCR distal C terminus. PDZ proteins can have robust effects on GPCR cellular localization, signal transduction coupling, ligand binding, and duration of action [4]. Since the seminal discoveries of inaA binding to rhodopsin [5] and NHERF binding to the β2-adrenergic receptor (AR) [6, 7], much effort has been put forth to expose GPCR:PDZ protein interactions and deconvolute their functional roles. Designing small molecules that alter a GPCR:PDZ protein interface may permit precise modulation of specific GPCR signaling pathways, while limiting unwanted side effects, more so if a GPCR:PDZ protein interaction is unique and cell-type specific. However, before this intriguing new field of pharmacology can be harvested, GPCR:PDZ protein interactions must be thoroughly characterized.

At least 30 human GPCRs contain a putative Type I PDZ ligand on the distal portion of their C-terminal tail with a conserved X-[S/T]-X-[ϕ] sequence [8]. GPCRs within this subfamily are diverse and include adrenergic (ADRA1D, ADRA2B, ADRB1, ADRB2); somatostatin (SSTR1-5); serotonin (HTR2A,B,C), chemokine (CXCR1,2,3,5); purinergic (P2RY1,12); and others (GALR1, HRH3, MCHR2, C3AR1, LPAR2, S1PR2). There appears to be no simple trend to categorize these receptors—they include Gαs-, Gαq- and Gαi-coupled GPCRs; some are stimulated by small molecules, others peptides, a few by lipids; and have distinct anatomical locations in the body where they regulate a plethora of physiological functions.

We previously discovered that certain members of the syntrophin family of PDZ domain proteins (SNTA, SNTB1, and SNTB2) bind the C-terminal Type I PDZ ligand of α_1D_-AR (ADRA1D) [9], a GPCR targeted in cardiovascular disease, benign prostatic hypertrophy, and urinary tract disorders [10]. Syntrophins facilitate ADRA1D plasma membrane expression and functional coupling by recruiting members of the dystrophin-associated protein complex (DAPC) [11, 12]. To assess the uniqueness of this GPCR complex and its potential to be selectively targeted with drugs, a panel of Type I PDZ ligand GPCRs was subjected to proteomic analysis. The results were surprising. The ADRA1D:syntrophin interaction is unique—none of the other Type I PDZ ligand GPCRs formed this complex. Of particular novelty, the ADRA1D binds a second PDZ domain protein simultaneously in living cells—Scribble (SCRIB), a multi-domain scaffold essential for cell polarity and migration.

## Results

### Interactomes of Type I PDZ ligand GPCRs

The uniqueness of the ADRA1D:syntrophin interaction was examined by subjecting 23 Type I PDZ ligand GPCRs to tandem affinity purification followed by mass spectrometry (TAP/MS). Table 1 provides a summary of the results (raw data in Supplementary Table S1). PDZ proteins detected included Golgi-associated PDZ and coiled-coiled motif-containing protein (GOPC) for SSTR1 and CXCR3, and PDZ domain-containing 8 (PDZD8) for GALR1. Of particular interest was CYSLTR2, which bound four PDZ domain proteins (CASK, LIN7C, DLG1, and MPP7), and MAS1, which bound two PDZ domain proteins (CASK and DLG1). Several unique non-PDZ interacting proteins were detected, as well as many non-specific interactors, presumably due to their high abundance in cells (that is, heat-shock proteins, ERAD/proteosome components, and ribosomal subunits). None of the GPCRs examined interacted with syntrophins, nor the DAPC, indicating the ADRA1D:syntrophin complex is unique among Type I PDZ ligand GPCRs.

To further define the ADRA1D complex, higher resolution analysis was used to optimize previously performed TAP/MS studies [11, 12]. Again, multiple members of the DAPC were identified: syntrophins (SNTB1, SNTB2), utrophin (UTRN), dystrophin (DMD), dystrobrevin-A (DTNA), and α-catulin (CTNNAL1). Unexpectedly, a second PDZ domain protein—Scribble (SCRIB)—was detected. Why was SCRIB not detected in previous studies? A re-examination of previous TAP purification procedures [11, 12] revealed increased bait protein coverage when TEV cleavage is omitted from the TAP protocol (ADRA1D: 15.4 vs 4%; DTNA 17.6 vs 6%; SNTB1: 27.9 vs 26%; SNTB2: 42.4 vs 19%; UTRN 17 vs 2%). Thus, SCRIB detection can be attributed to improved purification technique.

This result raised an intriguing question: does the ADRA1D exist in two distinct, unique complexes in cells, ADRA1D:syntrophin and ADRA1D:SCRIB, or as an ADRA1D:syntrophin:SCRIB macromolecular complex?

### Syntrophins and SCRIB compete for the ADRA1D PDZ ligand

**Figure 1:**
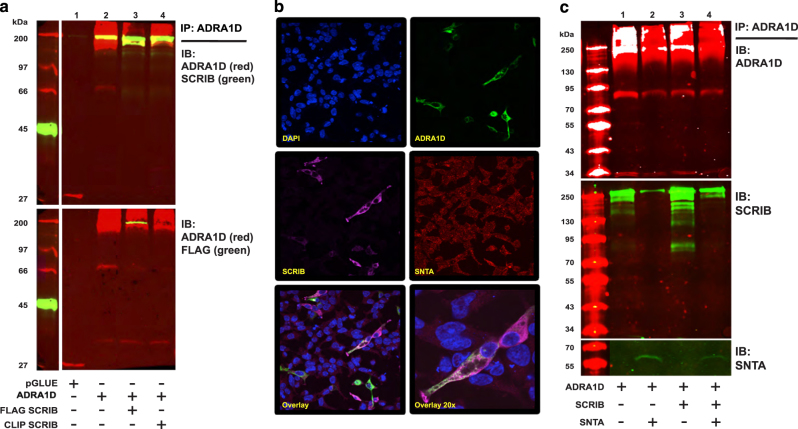
ADRA1D interacts with syntrophin and SCRIB. (**a**) TAP–ADRA1D co-purifies with FLAG- (bottom panel) and endogenous (top panel) SCRIB. (**b**) Confocal image displaying ADRA1D, SNTA, and SCRIB co-localization in HEK 293T cells. (**c**) SNTA and SCRIB compete for the ADRA1D PDZ ligand. Overexpressing SNTA competes away SCRIB:ADRA1D binding (lane 2). Co-overexpressing SNTA and SCRIB substantially diminishes SCRIB:ADRA1D binding (lane 4).

The ADRA1D:SCRIB interaction was further examined using affinity purification of ADRA1D followed by western blotting. ADRA1D interacted with both endogenous and ectopic SCRIB (Figure 1a, lanes 2 and 3, respectively), confirming the TAP/MS results. Furthermore, fluorescence microscopy studies indicate ADRA1D and SCRIB co-localize at the plasma membrane and within the cell (Figure 1b), establishing SCRIB as a novel interactor of ADRA1D.

As SCRIB and syntrophins are PDZ proteins, it is likely that they compete for the ADRA1D PDZ ligand. In support of this, SNTA significantly diminished ADRA1D:SCRIB formation (Figure 1c, lane 2), while co-expressing SCRIB and SNTA significantly diminished ADRA1D:SCRIB binding, and reduced the amount of ADRA1D:SNTA binding by ~50% as determined by band densitometry analysis (Figure 1c, lane 4). Altogether, these data indicate syntrophins and SCRIB compete for the ADRA1D PDZ ligand.

We next addressed whether ADRA1D:SCRIB and ADRA1D:syntrophin interactions are cell-type specific. TAP–ADRA1D was transfected into five additional cell lines of divaricating tissue origin, each containing similar SCRIB and syntrophin expression levels (Supplementary Figure S1), and then analyzed with TAP/MS (Table 2, Supplementary Table S2). DAPC members SNTB1, SNTB2, DMD, DTNA, and UTRN, as well as SCRIB, were detected in all five cell lines, suggesting this complex is conserved. Of particular interest, three additional PDZ proteins—DLG1, CASK, and LIN7A—were detected in SW480 colon cancer cells, suggesting additional, yet to be characterized, ADRA1D complexes exist and are cell-type dependent.

### Syntrophins and SCRIB impart divergent pharmacological properties to the ADRA1D macromolecular complex

**Figure 2:**
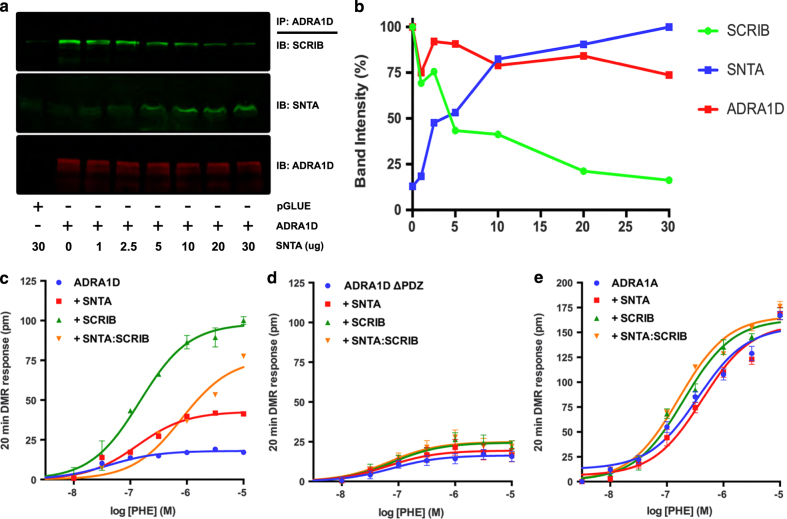
SNTA and SCRIB impart distinct ADRA1D pharmacological properties. (**a**) SNTA displaces SCRIB from the ADRA1D in a concentration-dependent manner. (**b**) Quantitation of SNTA and SCRIB association with ADRA1D in (**a**). Concentration-response curves for ADRA1D agonist phenylephrine (PHE)-stimulated DMR responses in HEK293T cells expressing TAP–ADRA1D (**c**), TAP–ADRA1D-ΔPDZ (**d**), or TAP-ADRA1A (**e**) alone, or co-expressing SNTA, SCRIB, or SNTA and SCRIB. Data are shown as mean±s.e.m., *n*=12–16.

The pharmacological properties of GPCR ligands can be altered by the cohort of proteins with which they interact [3]. Thus, we tested whether SCRIB and syntrophins differentially regulate the pharmacological properties of ADRA1D ligands by forcing the majority of ADRA1D into either an SNTA or SCRIB complex using cDNA titration. As shown, increasing the amount of transfected SNTA diminishes the SCRIB:ADRA1D interaction, but enhances the SNTA:ADRA1D interaction (Figure 2a, quantified in 2B).

We first tested whether SCRIB and SNTA regulate ADRA1D cell surface expression. Similar to our previous results, SNTA increased ADRA1D cell surface expression (Supplementary Figure S2). Likewise, SCRIB also increased ADRA1D surface expression (Supplementary Figure S2). We next measured the ADRA1D dynamic mass redistribution (DMR) responses to the agonist phenylephrine (PHE) with the Corning Epic BT. This high-throughput label-free signaling assay quantifies the sum of all signaling events downstream of receptor activation [13–15], and is advantageous in that it does not rely on a single, reductionist functional output. In the absence of SNTA or SCRIB, PHE stimulated minimal ADRA1D DMR responses (Figure 2c, Supplementary Figure S3), whereas SNTA induced a modest increase, in agreement with previous studies [11, 12]. PHE potency and intrinsic activity were greatest, however, when the majority of ADRA1D was bound to SCRIB. SNTA and/or SCRIB had no effect on PHE-stimulated DMR responses of ADRA1D missing the PDZ ligand (Figure 2d, Supplementary Figure S4) or the closely related ADRA1A subtype (Figure 2e, Supplementary Figure S5), which lacks a PDZ ligand, confirming the observed effects were specific to ADRA1D. Thus, syntrophins and SCRIB impart distinct functional properties that directly impact downstream ADRA1D signaling.

### Multimeric ADRA1D complexes contain both syntrophins and SCRIB

**Figure 3:**
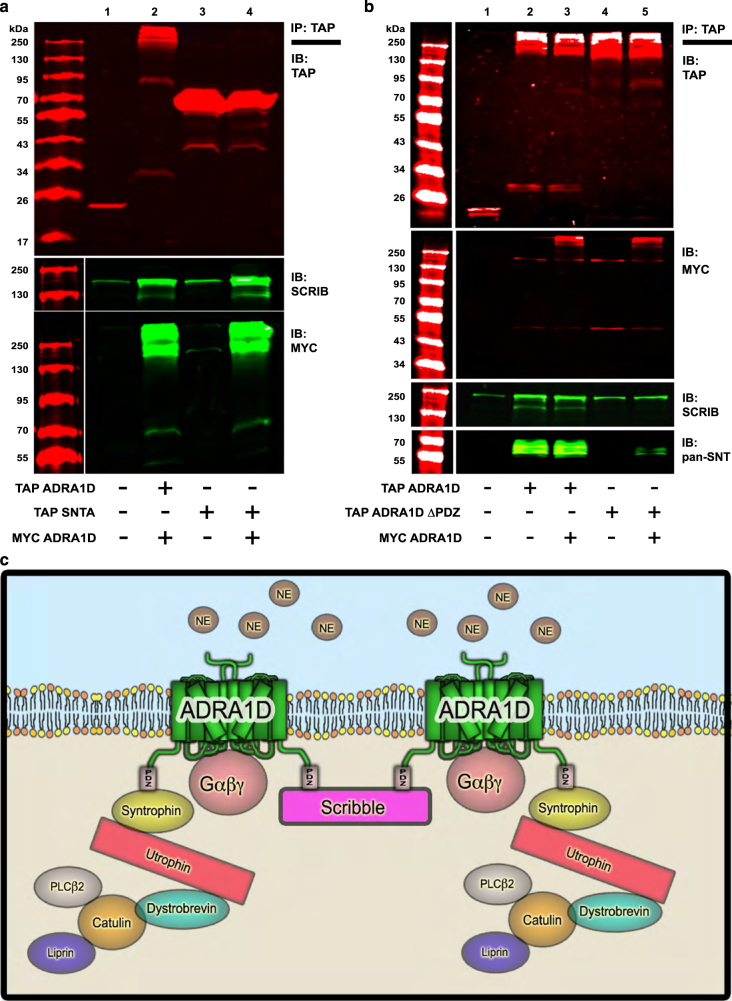
Syntrophins and SCRIB are contained within a single, multimeric ADRA1D complex. (**a**) TAP–SNTA co-purifies with SCRIB in the presence (lane 4), and not the absence (lane 3), of Myc–ADRA1D. (**b**) TAP–ADRA1D-ΔPDZ does not bind SCRIB or syntrophins (lane 4). Myc–ADRA1D:TAP–ADRA1D-ΔPDZ dimers selectively bind syntrophins and not SCRIB (lane 5). (**c**) Cartoon schematic of the multimeric ADRA1D:syntrophin:SCRIB complex.

Biochemical data show ADRA1D exists as a monomer at the expected size of ~79 kDa, though the majority of ADRA1D exists as dimers and/or higher order oligomers, as indicated by higher molecular weight bands (Figure 1c). Furthermore, ADRA1D interacts with at least two PDZ proteins, syntrophin and SCRIB, raising the possibility that syntrophins, SCRIB, and ADRA1D can exist in multiple complexes with unique stoichiometry, or in a single, multimeric ADRA1D complex. Biochemical analyses were used to assess these possibilities. Whereas SCRIB co-purified with TAP–ADRA1D (Figure 1a, lane 2; Figure 3a, lane 2), SCRIB did not co-purify with TAP-SNTA (Figure 3a, lane 3), proving syntrophins and SCRIB do not directly interact, and likely require ADRA1D to be present to be incorporated into the same complex. This was comfirmed by co-expressing Myc–ADRA1D, which facilitated SCRIB co-purification with TAP-SNTA (Figure 3a, lane 4), revealing that a SNTA-ADRA1D complex can recruit SCRIB. In parallel, SCRIB TAP/MS was performed in the absence or presence of ectopic ADRA1D (Table 3, Supplementary Table S3). In the absence of ADRA1D, TAP-SCRIB complexes contained known interactors, ARHGEF6/7, GIT1, and PPP1CCC, but not syntrophins or members of the DAPC. In the presence of ADRA1D, however, ADRA1D, DTNA, SNTB2, and UTRN were identified in TAP-SCRIB complexes, in addition to ARHGEF6/7, GIT1, and PPP1CCC. Thus, both proteomic and biochemical studies provide compelling evidence that SCRIB and syntrophins exist within a single ADRA1D macromolecular complex.

If syntrophin and SCRIB compete for the ADRA1D PDZ ligand, and are contained within a single ADRA1D complex, then ADRA1D must exist minimally as a homodimer, with at least two functional PDZ ligands. Veritably, both TAP–ADRA1D and TAP–ADRA1D-ΔPDZ can pull down Myc–ADRA1D (Figure 3b, lanes 3 and 5, respectively), demonstrating that ADRA1D dimers (and possibly multimers) are formed and are not dependent on the PDZ ligand. Given that syntrophins have a single PDZ domain, whereas SCRIB has four, the importance of having multiple ADRA1D PDZ ligands for complex assembly was tested. TAP–ADRA1D-ΔPDZ and Myc–ADRA1D were co-expressed and TAP–ADRA1D-ΔPDZ complexes were purified and analyzed by MS (Table 4, Supplementary Table S4). Any SCRIB or syntrophin interactions must occur through an ADRA1D dimer containing Myc–ADRA1D, as TAP–ADRA1D-ΔPDZ is unable to interact with either PDZ protein (Table 4, Supplementary Table S4). Both syntrophins and DAPC members were identified, whereas SCRIB was not.

Subsequent biochemical analyses demonstrate SCRIB and syntrophins co-purify with TAP–ADRA1D (Figure 3b, lane 2) and TAP–ADRA1D/Myc–ADRA1D complexes (Figure 3b, lane 3), but not with TAP–ADRA1D–ΔPDZ (Figure 3b, lane 4). When Myc–ADRA1D was co-expressed with TAP–ADRA1D–ΔPDZ, however, syntrophins co-purified with TAP–ADRA1D-ΔPDZ/Myc–ADRA1D complexes, whereas SCRIB did not (Figure 3b, lane 5). These findings clearly demonstrate syntrophins only require a single ADRA1D PDZ ligand, whereas SCRIB requires at least two.

Taken together, these findings indicate functional ADRA1D is expressed as homodimers/multimers in living cells, with one protomer bound to SCRIB and another to syntrophin (Figure 3c). Indeed, ADRA1D is mostly expressed as homodimers or higher-order oligomers, with only a fraction of the protein expressed as monomer (Figure 1b, lanes 2, 4; Figure 3b, lanes 3, 5). These results provide new information regarding the importance of GPCR homodimers, in that individual protomers are modular units capable of interacting with specific scaffolding proteins that recruit myriad subunits to the complex. In summary, we propose the ADRA1D exists as a dynamic, cell-context dependent, multimeric complex containing at least two PDZ proteins, SCRIB and syntrophins, and it is likely that other PDZ GPCRs (that is, Cys-LTR2, Mas1) demonstrate similar dimeric modular properties.

## Discussion

Identifying and characterizing GPCR macromolecular complexes provides opportunities to selectively target and modulate discrete signaling events with novel small molecules, moreso if the complexes are unique, and are expressed with cell-type specificity. Our initial purpose to perform these studies was to assess the uniqueness of the α_1D_-AR (ADRA1D):syntrophin complex via proteomic analysis of human GPCRs containing a Type I PDZ ligand. The results were clear—no other GPCRs examined associated with syntrophins. Unexpectedly, ADRA1D interacted with a 2nd PDZ protein, scribble (SCRIB), which again, no other GPCR interacted with. Thus, designing small molecules that disrupt the ADRA1D:syntrophin interaction, or enhance the ADRA1D:SCRIB interaction, may very well increase therapeutic efficacy over classical antagonists that block all downstream ADRA1D signaling events. The groundbreaking study identifying a β-arrestin2:Akt:protein phosphatase 2 complex as a target of lithium action in behavior disorders [16] provides compelling evidence that targeting GPCR signaling complexes with drugs is an untapped and fruitful area of drug discovery. In addition, the recent discovery of a patient with a P341A point mutation in the P2Y12 PDZ ligand that abrogates receptor function and induces bleeding disorder [17] conveys the clinical importance of identifying and characterizing GPCR:PDZ protein complexes.

Before individual ADRA1D:PDZ protein interactions can be targeted, their functional roles in cells must be deconvoluted. The purpose of syntophins for ADRA1D function is more established; syntrophins are multi-domain scaffolds that link membrane proteins—such as aquaporins, nicotinic acetylcholine receptors and neuronal nitric oxide synthase [18, 19]—to the DAPC, imparting functionality and localization to specific cellular microdomains. Such is the case for the ADRA1D, where loss of syntrophin causes ER retention and minimal functional responses [9, 11, 12]. The DAPC may also link GPCRs to non G-protein signaling events. Recruited by DTNA to the ADRA1D, CTNNAL1 can stimulate Lbc:Rho:ROCK [20], possibly to regulate cell migration—a physiological event regulated by ADRA1D in the peripheral vasculature during the process of vascular remodeling/restenosis following injury [21].

The role of SCRIB in the ADRA1D complex remains unclear. It is well recognized that SCRIB forms multiple protein complexes critical for embryonic development [22]. In *D. melanogaster*, for example, the Scrib: lethal giant larvae: discs large complex regulates cell polarity and growth [23], whereas in human cells, SCRIB complexes with AHRGEF:PAK1:GIT1, or NOS1AP:VANGL2, to regulate cell invasion and migration [24, 25]. In each case, SCRIB is thought to ensure proper cellular localization of the complex. This may also be true for the ADRA1D. SCRIB PDZ domain 2 recognizes PDZ ligand X-ET-X-V, whereas the remaining three domains recognize X-ET-X-ϕ [26]. The ADRA1D PDZ ligand, RETDI, should, in theory, be recognized by all four SCRIB PDZ domains. If true, SCRIB could potentially bind up to four ADRA1D protomers, permitting a second ADRA1D protomer in the homodimer/multimer to bind another SCRIB, or the syntrophin:DAPC (Figure 3c). ADRA1D has been long known to have a pivotal role in numerous physiological processes, including the cardiovascular system, where it regulates vascular tone of peripheral blood vessels [10], particularly the aorta [27] and coronary arteries [28], as well as contraction of the prostate [29, 30] and urinary vessels [31]. Given SCRIB is ubiquitously expressed, and ADRA1D interacted with SCRIB in all five cell lines examined in this study, it is reasonable to assume that ADRA1D and SCRIB form complexes in physiologically relevant tissues such as prostate, ureter, aorta, and coronary arteries, for the purpose of clustering ADRA1D in discrete signaling microdomains adjacent to sympathetic nerves, where they can be bombarded with salvos of secreted norepinephrine.

How GPCRs are structurally organized in cells remains a topic of high interest. Cell-based functional [32] and crystal structure [33] studies indicate GPCR dimers are likely formed, though parallel studies clearly demonstrate a GPCR monomer can bind ligand and signal [34, 35]. Elegant FRET-BRET studies performed on the D2-dopamine receptor in living cells provide convincing evidence for the existence of GPCRs in groups of four [36]. Our study provides additional evidence that GPCRs are simultaneously expressed as monomers, homodimers, and multimers in a cell at any given time. We speculate that the majority of ADRA1D exists as homodimers/multimers, based on our biochemical data, and that the PDZ ligand is not essential for dimer formation. Previous studies suggest dimerization occurs via interactions between the α-helical transmembrane domains 1 and 8 [37, 38], or possibly 4, 5, and 6 [39, 40]. However, the specific residues necessary for ADRA1D dimers to form have yet to be determined. Taken together, this emerging area of GPCR biology should be heavily considered when searching for novel drugs targeting GPCRs containing a PDZ ligand, as the PDZ proteins engaged in a specific GPCR macromolecular complex may be cell-type dependent, dynamic, and potentially impart unique pharmacological and signal transduction properties.

## Materials and Methods

### Plasmids, chemicals, and antibodies

Human Type I PDZ GPCR cDNAs were purchased from the Missouri S&T cDNA resource center (cdna.org) or cloned from human brain cDNA library (provided by Professor Ning Zheng, HHMI, University of Washington Department of Pharmacology). Human α-syntrophin cDNA was purchased from OriGene Technologies, (Rockville, MD, USA). Human FLAG-Scribble was provided by Professor Jon Huibregtse (Department of Molecular Sciences, University of Texas at Austin). cDNAs were subcloned into pGlue (provided by Professor Randall T. Moon, HHMI, University of Washington Department of Pharmacology) using In-Fusion HD cloning technology (Clontech).

(R)-(−)-phenylephrine hydrochloride (P6126) was purchased from Sigma (St Louis, MO, USA). Anti-FLAG antibodies were purchased from Sigma (2368). Alexa fluor 633 goat-anti Rabbit IgG (A-21070) and Alexa fluor 568 Goat anti-rat (A-11011) antibodies from Life Technologies. Topro-3 iodide (T3605) is from Life Technologies. Rabbit polyclonal α-syntrophin (H-65, sc-50460), and rabbit polyclonal Scrib (H-300, sc-28737) antibodies from Santa Cruz Biotechnology. Mouse monoclonal Anti-syntrophin (1351, ab11425) antibody and rabbit polyclonal anti-Myc tag antibody (ab9106) from Abcam (Cambridge, MA, USA). Anti-HA mouse mAb (6E2, #2367) from Cell Signaling (Danvers, MA, USA). IRdye 680 goat antimouse IgG and IRdye 800cw goat antirabbit IgG from Li-Cor (Lincoln, NE, USA).

### Cell culture and transfection

Human embryonic kidney (HEK) 293 T, HeLa, A549, MCF-7, A375, and SW480 cells were grown in Dulbecco’s modified Eagle’s medium (DMEM) supplemented with 10% fetal bovine serum and 2 mM L-glutamine. Cells were transfected with 1 mg ml^−1^ polyethylenimine and used 24–48 h post transfection.

### Confocal imaging

HEK293T cells were plated on cover slips at ~1.9×10^5^ cells per sample. Cells were transfected with cDNA constructs:polyethylenimine for 48 h and fixed with 4% PFA for 30 min. Cells were washed 3× with PBS, and incubated with blocking buffer containing 0.2% BSA/5% goat serum. When permeabilizing cells, 0.1% Triton X was included in the buffer. Cover slips were incubated with primary antibodies (Anti-HA 1:300; Anti-FLAG 1:100; anti-SNTA 1:500) overnight at 4 °C in blocking buffer, washed 3× with PBS, and for 1 h with secondary Alexa-fluor antibodies (1:1 000) in blocking buffer. Cover slips were mounted with Prolong Gold mounting media (Invitrogen, Grand Island, NY, USA) and incubated for 24 h. Slides were examined on a Leica SL confocal microscope in the W.M. Keck Imaging Center (University of Washington).

### SNAP-cell surface staining

HEK293 cells were seeded in six-well plates at 8×10^5^ cells per well. Cells were transfected with SNAP-tagged cDNA constructs/polyethylenimine and replated in 96-well black optical bottom cell culture plates. Cell density was~90% confluency before assay commencement. SNAP-Surface 782 substrate was diluted in DMEM to designated concentrations and incubated at 37 °C/5% CO_2_ for 30 min. Cells were washed, fixed with 4% paraformaldehyde, then incubated with 1:10 000 nuclear stain TOPRO-3 to normalize for cell number. Plates were analyzed with a Li-Cor Odyssey Scanner (Li-Cor Biotechnology) and signal intensity quantified.

### Tandem affinity purification-mass Spectrometry

TAP purification of all constructs has been described previously [8, 9]. Eluates were directly analyzed on a Velos-Pro Orbitrap Elite hybrid mass spectrometer (Thermo Fisher, Waltham, MA, USA). Raw MS data were searched with SEQUEST (Thermo Fisher) or COMET [41], and protein false discovery rate was set at 2.5%.

### Affinity purification/immunoblotting

Cells were harvested and lysed in 0.5% digitonin, 75 mM Tris (pH 8.0), 2 mM EDTA, 5 mM MgCl_2_, 1 mM DTT with protease and phosphatase inhibitors. Cleared supernatants were then subjected to affinity purification with streptavidin sepharose (GE Healthcare, Pittsburgh, PA, USA) for 3 h at 4 °C. Streptavidin beads were washed 4× with lysis buffer. Samples were denatured by boiling in 4× sample buffer at 90 °C for 10 min. Gels were transferred to nitrocellulose membrane and blocked for 1 h at room temperature and then probed with primary antibodies as indicated overnight at 4 °C. Primary antibodies were detected with IRDye 800CW goat anti-rabbit or IRDye 680 goat anti-mouse and imaged with a LiCor Odyssey Scanner (LiCor Biotechnology). Of note, a faint band of similar molecular weight to SCRIB was consistently detected in anti-SCRIB blots (Figure 1c, lane 2; Figure 3a, Lanes 1 and 3; Figure 3b, lanes 1, 4, 5). However, TAP/MS with TAP–ADRA1D–ΔPDZ confirmed this band is not SCRIB (Table 4).

### Dynamic mass redistribution assays

HEK293T cells were seeded at ~500 k per well in 384-well Corning Epic sensor microplates and cultured for 24 h. Cells were washed 3× with HBSS buffer and transferred to a Corning Epic BT reader at 37 °C. Baseline DMR measurements were recorded for 1 h. Compounds were added with the Sorenson Biosciences 96-well Benchtop Pipettor and agonist DMR responses were recorded. Data were exported to Microsoft Excel using Epic Analyzer Software (Corning, NY, USA).

### Data analysis

Data were analyzed with GraphPad Prism 6 software (La Jolla, CA, USA).

## Figures and Tables

**Table 1:** Proteomic analysis of Type I PDZ GPCRs in mammalian cells

*GPCR*	*%Cov*	*#UP*	*PDZ*	*%Cov*	*#UP*	*Non-PDZ*	*%Cov*	*#UP*
ADRA1D	15.4	9	SCRIB	10.7	12	CTNNAL1	1.1	1
			SNTB1	27.9	11	DMD	3	10
			SNTB2	42.4	19	DTNA	17.6	5
						UTRN	17	50
ADRA2B	33.2	6	a			a		
HTR2A	21.4	12	a			WRNIP1	2.1	1
HTR2B	21.8	17	a			WRNIP1	5	3
HTR2C	21.4	16	a			a		
SSTR1	16.9	8	GOPC	5.7	2	a		
SSTR2	10.6	4	a			a		
SSTR3	20.1	10	a			CSNK2A1	8.8	2
			a			GOLPH3	9.7	2
SSTR4	17	6	a			YWHAB	34	5
			a			YWHAE	35.6	6
			a			YWHAH	22	3
SSTR5	15.9	5	a			a		
CXCR1	22.3	6	a			a		
CXCR2	22.8	10	a			FAAH	7.8	2
CXCR3	11.4	3	GOPC	7.7	3	GHDC	11.2	5
CXCR5	26.1	11	a			GHDC	7.2	3
GALR1	12.6	6	PDZD8	1	1	a		
HRH3	25.5	7	a			a		
P2RY1	23.9	11	a			a		
P2RY12	27.8	8	a			a		
MCHR2	4.7	2	a			a		
C3AR1	26.1	9	a			a		
LPAR2	21.4	10	a			a		
S1PR2	19	8	a			a		
CYSLTR2	13.3	6	CASK	30.9	28	PLD3	4.3	2
			DLG1	25.5	20			
			LIN7C	37.6	7			
			MPP7	25.2	14			
MAS1	10.5	5	CASK	8.1	3	PPP6R1	10.1	2
			DLG1	25	10			

Abbreviations: ADRA1D, α_1D_ adrenergic; ADRA2B, ɑ_2B_-adrenergic; CXCR, chemokine; CYSLTR2, cysteinyl leukotriene 2; C3AR1, C3a anaphylatoxin chemotactic; GALR, galanin type I; HRH3, histamine type 3; HTR2, 5-hydroxytryptamine type 2; LPAR2, lysophosphatidic acid type 2; MAS1, proto-oncogene MAS type I; MCHR2, melanin-concentrating hormone type 2; P2YR, P2Y purinergic; SSTR, somatostatin; S1PR2, sphingosine-1-phosphate type 2s.

Data shown include GPCR bait, percent peptide coverage (%Cov), number of unique peptides (#UP), PDZ proteins (PDZ), and non-PDZ proteins (Non-PDZ) detected.

a No proteins detected.

**Table 2:** ADRA1D proteomic analysis in different human cell lines

*Cell type*	*ADRA1D*		*PDZ proteins*			*Non-PDZ proteins*		
*tissue*	*%Cov*	*#UP*	*Name*	*%Cov*	*#UP*	*Name*	*%Cov*	*#UP*
HEK293T	15.4	9	SCRIB	10.7	12	CTNNAL1	1.1	1
kidney			SNTB1	27.9	11	DMD	3	10
			SNTB2	42.4	19	DTNA	17.6	5
						UTRN	17	50
HeLa	10.0	4	SCRIB	13.5	8	CTNNAL1	13.6	8
cervix			SNTA	6.3	4	DTNA	4	9
			SNTB1	27.7	13	DTNB	17.3	8
			SNTB2	37.6	26	UTRN	20.3	62
A549	2.6	2	SCRIB	2.3	2	DMD	8.5	4
lung			SNTB2	32.4	17	DTNB	6.3	4
						MAPK1	4.7	2
						UTRN	11.3	36
MCF-7	9.8	3	SCRIB	4.9	6	DMD	7.4	4
breast			SNTB2	27.4	15	DTNA	26.1	9
						DTNB	7.9	5
						MAPK1	9.2	4
						MAPK3	9.8	3
						UTRN	15.7	48
A375	2.6	2	SCRIB	2.8	2	DMD	4.5	2
skin			SNTB2	9.6	28	DTNA	8.7	4
						DTNB	5	3
						UTRN	9.6	28
SW480	8.6	5	CASK	5.1	4	CTNNAL1	10.9	7
colon			DLG1	7.2	5	DTNA	11	3
			LIN7A	7.7	1	DTNB	17.1	9
			SCRIB	8.8	12	PPFIA1	11.1	8
			SNTB1	37	20	PPFIBP1	12.9	9
			SNTB2	43.9	26	UTRN	22.5	76

Data shown include cell type and source tissue, percent peptide coverage (%Cov), number of unique peptides (#UP), PDZ and non-PDZ proteins detected.

**Table 3:** SCRIB proteomic analysis in the absence and presence of ADRA1D

*EXP*	*SCRIB*		*SCRIB interactors*			*ADRA1D interactors*		
*TAP-SCRIB*	*%Cov*	*#UP*	*Name*	*%Cov*	*#UP*	*Name*	*%Cov*	*#UP*
+ Empty vector	58.1	137	ARHGEF6	5.5	11	a	a	a
			ARHGEF7	19.8	7			
			GIT1	13.5	6			
			PPP1CC	31.8	2			
+ ADRA1D	59.1	134	ARHGEF6	18.3	8	ADRA1D	26.2	6
			ARHGEF7	24.3	11	DTNA	3.5	1
			GIT1	42.6	24	SNTB2	4.9	1
			PPP1CC	18.5	5	UTRN	1.3	4
+ ADRA1D–ΔPDZ	57.8	137	ARHGEF6	18	6	a	a	a
			ARHGEF7	23.7	11			
			GIT1	36.9	21			
			PPP1CC	18.5	5			

HEK293T cell lysates expressing SCRIB alone (+ empty vector), + WT α_1D_-AR (+ ADRA1D), or ΔPDZ α_1D_-AR (+ ADRA1D–ΔPDZ) were subjected to TAP/MS. Shown are % protein coverage (%Cov) and number of unique peptides (#UP).

a No proteins detected.

**Table 4:** Multimeric ADRA1D proteomic analysis

*EXP*	*ADRA1D*		*PDZ proteins*			*Non-PDZ proteins*		
	*%Cov*	*#UP*	*Name*	*%Cov*	*#UP*	*Name*	*%Cov*	*#UP*
TAP–ADRA1D	15.4	9	SCRIB	10.7	12	CTNNAL1	1.1	1
			SNTB1	27.9	11	DMD	3	10
			SNTB2	42.4	19	DTNA	17.6	5
						UTRN	17	50
TAP−ADRA1D–ΔPDZ	12.8	3	a	a	a	a	a	a
TAP−ADRA1D–ΔPDZ	17.8	10	SCRIB	a	a	CTNNAL1	a	a
+ Myc–ADRA1D			SNTB1	12.8	4	DMD	8.4	3
			SNTB2	37	16	DTNA	14.4	2
						UTRN	8.8	21

HEK293T cell lysates expressing WT (TAP–ADRA1D), ΔPDZ (TAP–ADRA1D–ΔPDZ) α_1D_-AR, or TAP–ADRA1D–ΔPDZ co-transfected with MYC α_1D_-AR (Myc–ADRA1D) were subjected to TAP/MS. Shown are % protein coverage (%Cov), number of unique peptides (#UP) for PDZ and non-PDZ proteins.

a No proteins detected.
