# Supplementary figures and images for: Individual protomers of a G protein-coupled receptor dimer integrate distinct functional modules
Source: Cell Discov. 2015 Jun 16;1:15011–. doi: 10.1038/celldisc.2015.11 (PMC4658663; doi:10.1038/celldisc.2015.11)

Figure S1

HEK    HeLa    SW480    MCF-7    A375    A549

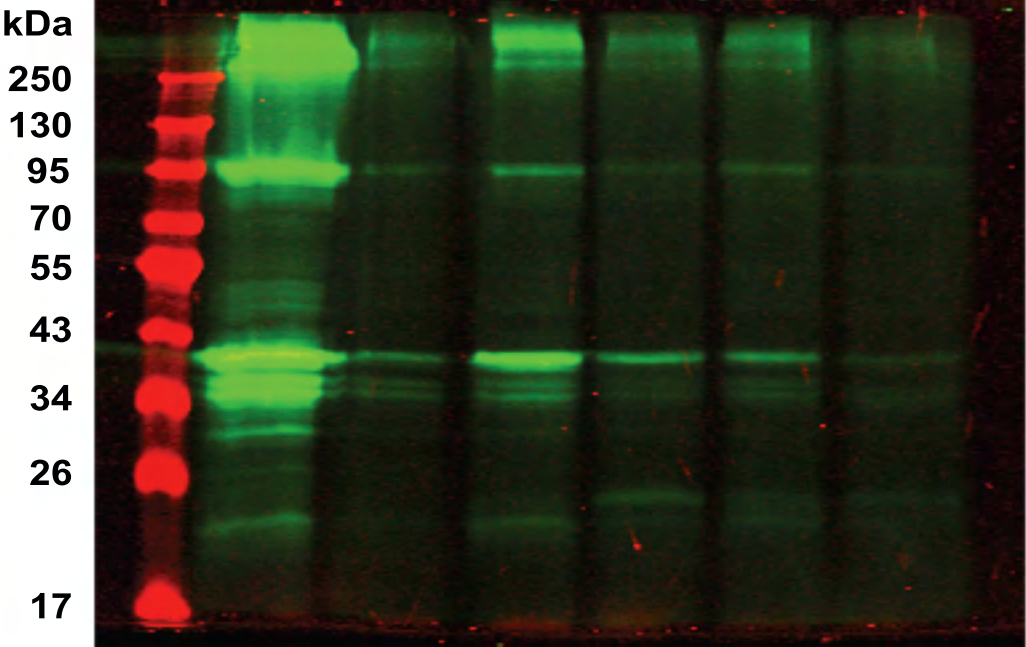

IB:  
ADRA1D

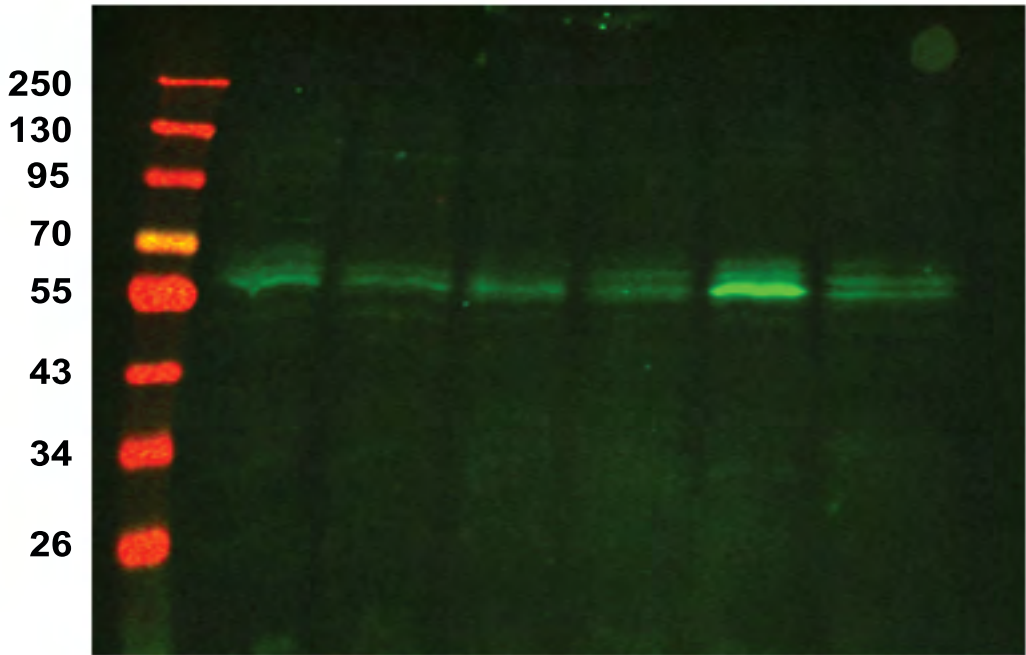

IB:  
pan-SNT

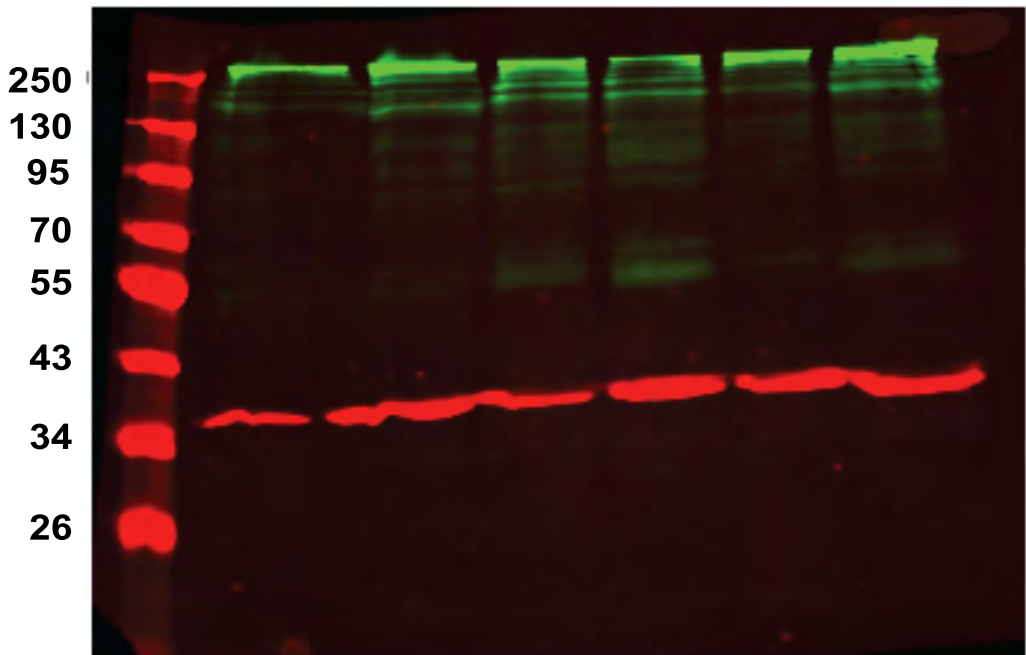

IB:  
SCRIB (green)  
GAPDH (red)

Supplement: Supplementary Figure S1 [file celldisc201511-s1.pdf]

**Figure S2**

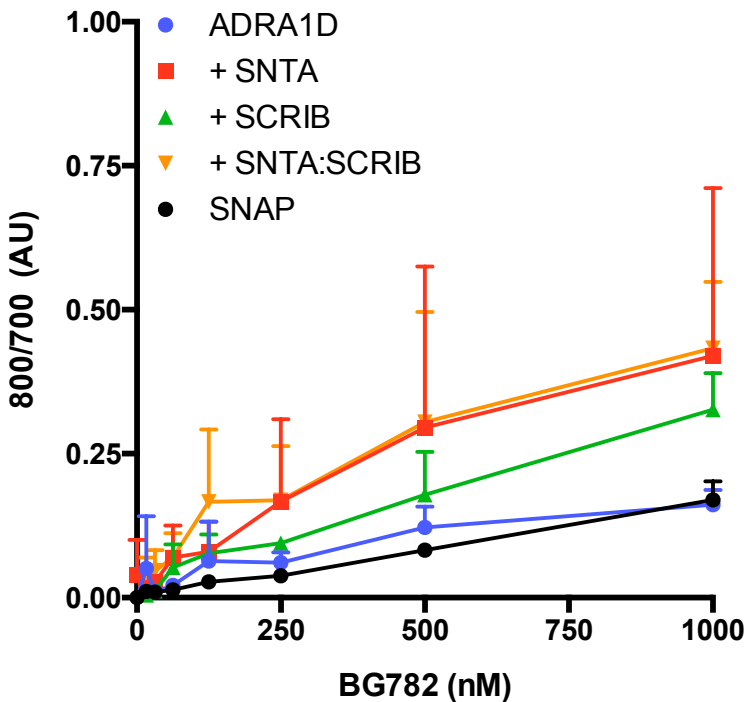

Supplement: Supplementary Figure S2 [file celldisc201511-s2.pdf]

Figure S3

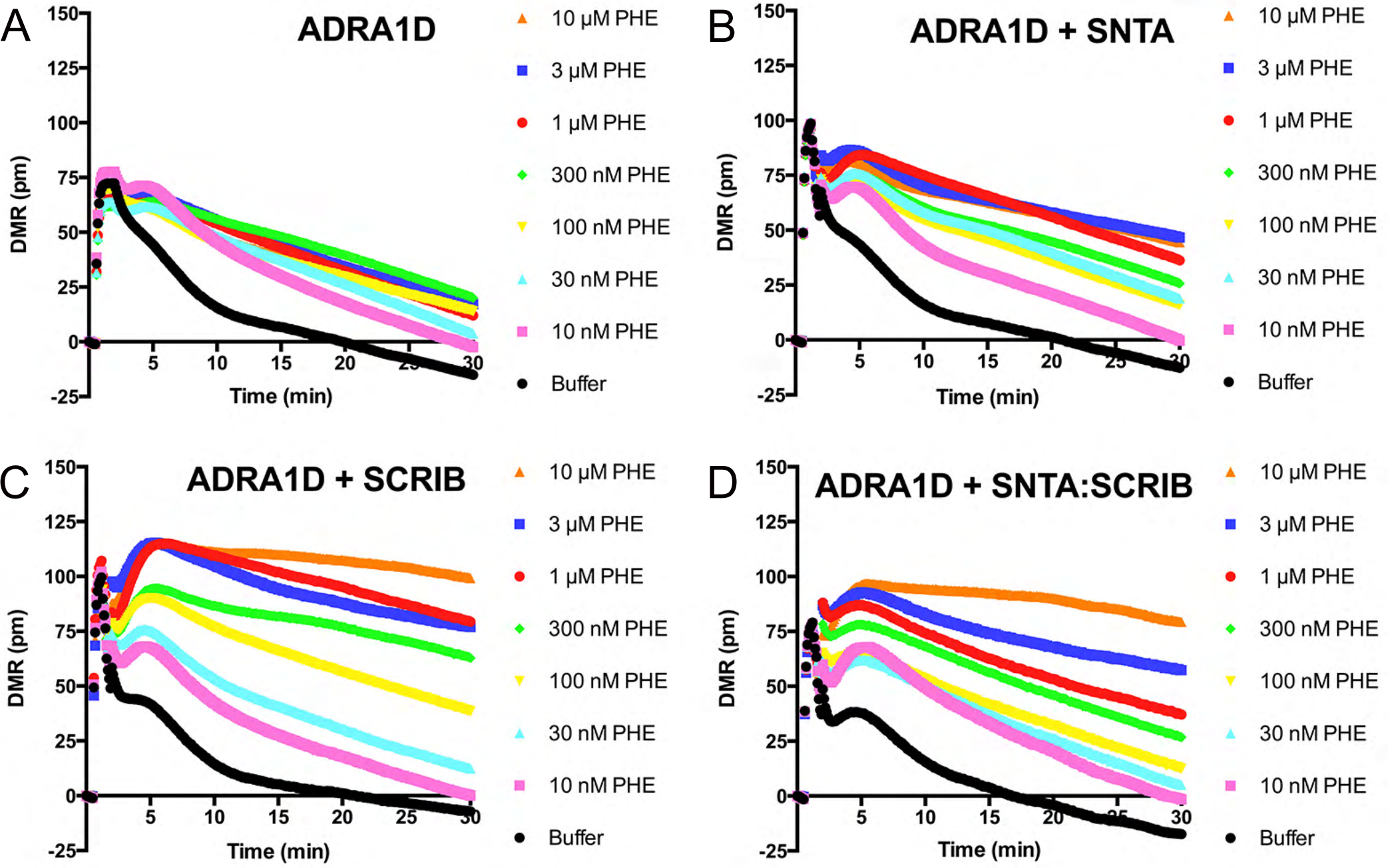

Supplement: Supplementary Figure S3 [file celldisc201511-s3.pdf]

**Figure S4**

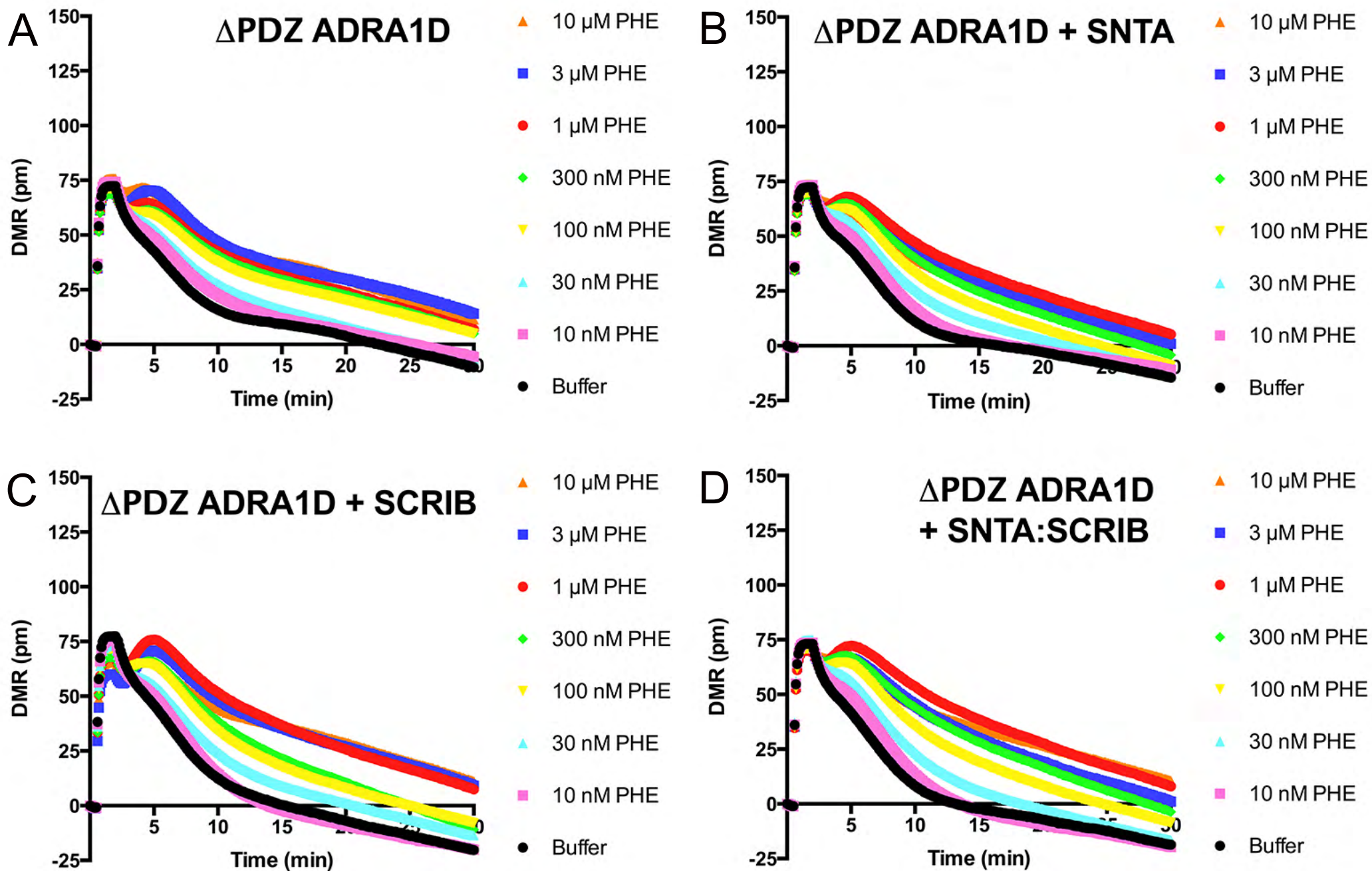

Supplement: Supplementary Figure S4 [file celldisc201511-s4.pdf]

**Figure S5**

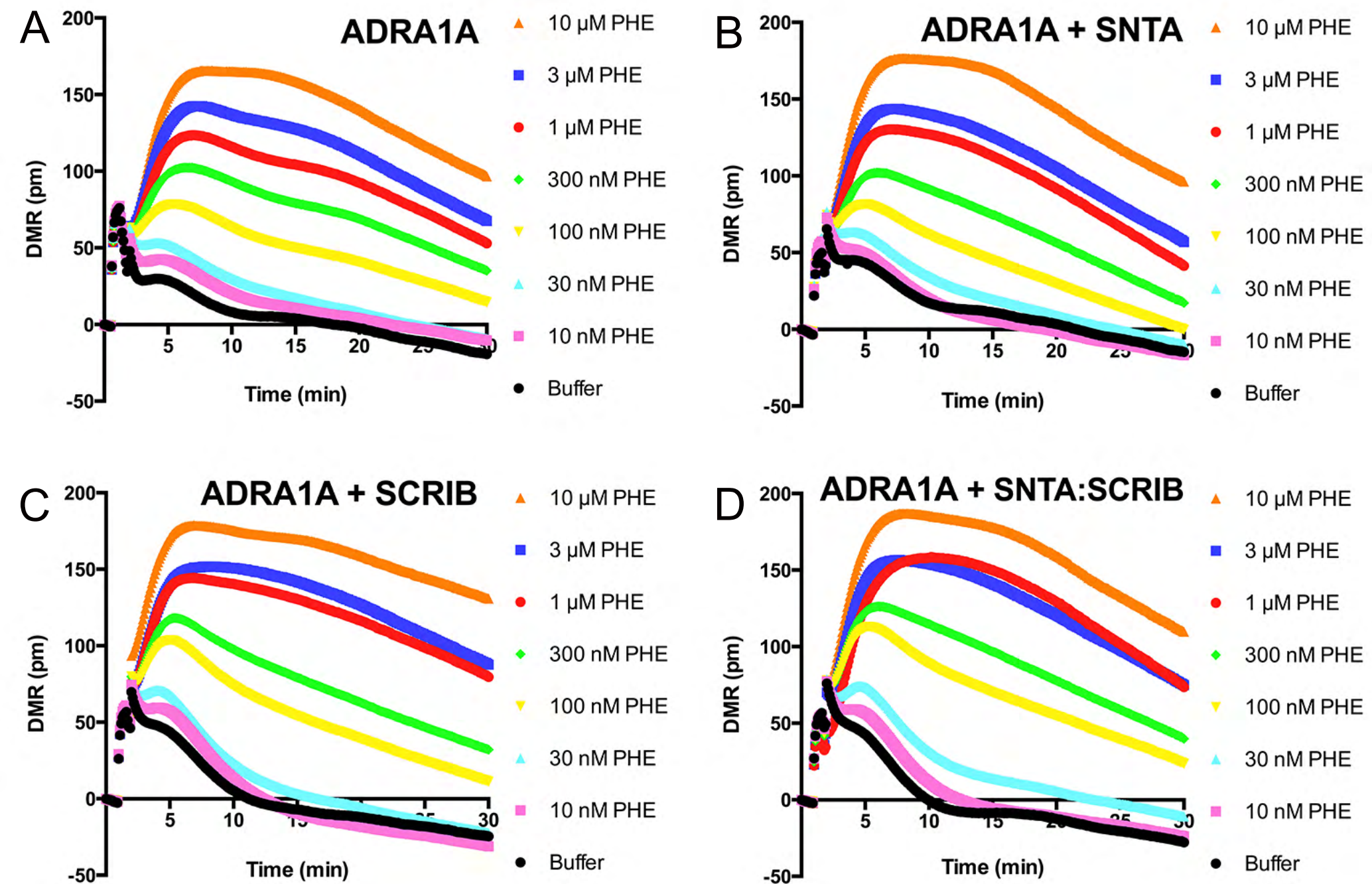

Supplement: Supplementary Figure S5 [file celldisc201511-s5.pdf]
