# Supplementary material for: Individual protomers of a G protein-coupled receptor dimer integrate distinct functional modules
Source: Cell Discov. 2015 Jun 16;1:15011–. doi: 10.1038/celldisc.2015.11 (PMC4658663; doi:10.1038/celldisc.2015.11)
Supplement: Supplementary Figure and Table Legends [file celldisc201511-s10.pdf]

Fig. S1. Protein expression levels in cell lines used for TAP-ADRA1D proteomic screens. Shown are SNAP-ADRA1D (upper panel), syntrophins (pan-SNT, middle panel) and SCRIB (green, lower panel) or GAPDH (red, lower panel).

Fig. S2. SNAP cell-surface staining of ADRA1D in the absence and presence of SNTA, SCRIB or SNTA:SCRIB. HEK cells were plated, transfected with cDNA constructs as indicated, and treated with increasing concentrations of cell impermeable SNAP substrate BG-782. To-PRO-3 nuclear stain was used to normalized for cell density. SNAP-ADRA1D cell surface expression was quantified as the ratio of ADRA1D/To-PRO-3 nuclear stain in arbitrary fluorescence units (800/700 AU). Data 584 are mean ± SEM, n =3.

Fig. S3. Effects of SNTA and SCRIB on ADRA1D functional responses. Phenylephrine (PHE) stimulated DMR responses were quantified for TAP-ADRA1D alone (A), + SNTA (B), + SCRIB (C), or + SNTA/SCRIB (D). Data are mean ± SEM, n = 12 - 16.

Fig. S4. Effects of SNTA and SCRIB on ΔPDZ ADRA1D functional responses. Phenylephrine (PHE) stimulated DMR responses were quantified for TAP-ADRA1D PDZ ligand truncated (ΔPDZ) alone (A), + SNTA (B), + SCRIB (C), or + SNTA/SCRIB (D). Data are mean ± SEM, n = 12 - 16.

Fig. S5. Effects of SNTA and SCRIB on ADRA1A functional responses. Phenylephrine (PHE) stimulated DMR responses were quantified for a non-PDZ GPCR, ADRA1A. Shown are TAP-ADRA1A alone (A), + SNTA (B), + SCRIB (C), or + SNTA/SCRIB (D). Data are mean ± SEM, n = 12 - 16.

Table S1. Proteomic datasets from Type I PDZ GPCR TAP/MS screens. 559 Peptide hits for each Type I PDZ GPCR are shown in sequential tabs. The Table of Contents (TOC) in Tab 1 contains hyperlinks to each sheet.

Table S2. Proteomic datasets from TAP-ADRA1D cell-type specific TAP/MS screens. Experiments are shown in sequential tabs. The Table of Contents (TOC) in Tab 1 contains hyperlinks to each sheet.

Table S3. Proteomic datasets from TAP-SCRIB TAP/MS screens. Experiments are shown in sequential tabs. The Table of Contents (TOC) in Tab 1 contains hyperlinks to each sheet.

Table S4. Proteomic datasets from TAP-ADRA1D multimer TAP/MS screens. Experiments are shown in sequential tabs. The Table of Contents (TOC) in Tab 1 contains hyperlinks to each sheet.
